# Seedling ectomycorrhization is central to conifer forest restoration: a case study from Kashmir Himalaya

**DOI:** 10.1038/s41598-022-17073-7

**Published:** 2022-08-03

**Authors:** Rezwana Assad, Zafar Ahmad Reshi, Irfan Rashid

**Affiliations:** Department of Botany, University of Kashmir, Srinagar, Jammu and Kashmir 190006 India

**Keywords:** Ecology, Plant sciences, Ecology, Environmental sciences

## Abstract

Over the past few decades, many countries have attempted to carry out forest landscape restoration over millions of hectares of degraded land. Such efforts, however, have met with limited success because of several factors, including a lack of adequate emphasis on ectomycorrhization of the nursery seedlings. A similar scenario is seen in the Kashmir Himalaya, where the natural regeneration of degraded forests is poor despite ample restoration efforts by forest managers. To overcome this challenge, we identified two promising ectomycorrhizal species, namely *Clitocybe nuda* and *Cortinarius distans*, for their use in ectomycorrhization of seedlings of three common conifers, namely *Abies pindrow*, *Cedrus deodara*, and *Picea smithiana*. Laboratory studies were carried out to investigate the requirements for optimum mycelial growth of these ectomycorrhizal fungi. Best ECM mycelial growth was obtained in the basic MMN medium containing glucose as the source of carbon and nitrogen in ammonium form. *C. distans* showed higher growth than *C. nuda* across all the treatments and also proved significantly more effective in enhancing the survival and growth of the conifer host plant seedlings. The present study resulted in standardizing the requirements for mass inoculum production of the two mycobionts which could help in successful forest restoration programmes.

## Introduction

Degradation of forest ecosystems due to detrimental anthropogenic activities poses a severe threat to biodiversity, impeding forest regeneration and impairing invaluable ecosystem services^1,2^. Maintenance and restoration of structural organization and functional integrity of forest ecosystems is critical global precedence^2–4^. The practice currently adopted for the restoration purpose is planting of conifer seedlings in the degraded forest ecosystems^5,6^. However, it has been reported that the survival of these planted seedlings in the disturbed and degraded forest lands is very low^7^ and hence it constrains the success of such restoration projects. Moreover, experiences worldwide reveal that degraded land restoration projects achieve little success or eventually fail altogether^8^. Hence, understanding the underlying causes and accordingly devising appropriate restoration strategies is crucial. In doing so, one of the often-overlooked reasons for these restoration failures is the inadequate mycorrhization of the nursery seedlings.

Ectomycorrhizal fungi (ECM) are a ‘keystone’ element of a functional community and one of the most effective biological tools for forest restoration projects^9^. Successful reforestation and restoration depends on the early capture of site resources by tree seedlings which, in turn, assure space, a continuing resource supply and vigour to resist pests, pathogens and climate stress^10,11^. Ectomycorrhizal fungi, in this context, are the key to optimal establishment and performance of forest tree species both under nursery and outplanted conditions^12–15^. Hence use of ECM fungi in ectomycorrhization of conifer seedlings is of critical importance in ensuring that the planted seedlings survive, grow and establish under normal as well as stressful conditions.

ECM fungi persist only for a short duration after disturbance. Generally, they fail to survive in the soil for prolonged periods without being associated with the roots of a living host^16^, except for some disturbance-resistant suilloid fungi like *Rhizopogon* and *Suillus* species which become dominant after disturbance^17^. The post-disturbance natural recovery of ECM symbionts is a sluggish progression and generally takes decades to return to its original position^18^. Consequently, highly disturbed forests lack requisite ectomycorrhizal association on plant root systems, which is one of the main reasons for high seedling mortality, poor regeneration, or even complete regeneration failure in such areas^19,20^. A potential eco-friendly approach to overcome this regeneration failure is controlled ectomycorrhization by pre-inoculating the conifer seedlings with markedly adapted ECM fungi for their successful re-establishment and prospective use in large-scale reforestation projects^15,21,22^. Nevertheless, apt selection of naturally-associated suitable host-mycobiont, which is best adapted to the ultimate planting location, is crucial for mycorrhization^23^. Thus, effective restoration of degraded forests and other allied ecosystems can be premeditated only when ample information is available regarding the compatibility and efficiency of ECM fungal symbionts associated with the target host plants^24^. Several studies involving in vitro cultivation of ectomycorrhizal fungi have been undertaken to standardize the conditions for optimal inoculum production^25–29^, which is a prerequisite for large scale ectomycorrhization.

ECM fungi are most commonly isolated from fruiting bodies due to their ease of identification, and the resultant vegetative mycelia have been proved to be the most effective form of inoculum for ectomycorrhization of host plant seedlings^30^. However, the synthesis, establishment, growth, function and symbiotic alliance of ectomycorrhizal fungi is highly dependent on a range of biotic and abiotic factors and their interactions in natural as well as under laboratory conditions^28,29^. Among these factors, nutrient availability, predominantly that of carbon (C) and nitrogen (N) sources, is of immense significance^27–29,31^.

The present investigation was undertaken to study the growth response of two ectomycorrhizal fungi (*Clitocybe nuda* and *Cortinarius distans*) to various C and N sources under in vitro culture conditions to determine their requirements for optimum mycelial growth, necessary for the production of sufficient inoculum required for subsequent utilization in ectomycorrhization of conifer seedlings.

As the Kashmir Himalayan forests have constantly undergone unprecedented deforestation and degradation for the past few decades, and the projected estimates of forest cover for 2030 have demonstrated that there will be a further increase in forest degradation^32^. In this backdrop, the present study was also aimed to assess the effectiveness of ectomycorrhization on the seedling survival and growth of three dominant conifers of Kashmir Himalayan forests, namely *Abies pindrow* (Royle ex D.Don) Royle (Himalayan Pindrow Fir), *Cedrus deodara* (Roxb. ex D.Don) G.Don (Himalayan Cedar) and *Picea smithiana* (Wall.) Boiss. (West Himalayan Spruce). This study explicitly addresses whether the two selected ECM fungi exhibit the capability to form an association with the seedlings of the three pinaceae host plant species and highlights the effect of ectomycorrhization on the performance of conifer seedlings for their potential use in prospective restoration practices of degraded forest ecosystems.

## Materials and methods

### Fungal species studied

During the present exploration, two ECM fungi viz., *Clitocybe nuda* (Bull.) H.E. Bigelow & A.H. Sm. and *Cortinarius distans* Peck, were found predominantly associated with the conifers in the Kashmir Himalayan forests. The rationale for selecting these two particular ECM species was that besides being allied with the conifers globally^33,34^, these two ECM fungi were reported to successfully promote growth and survival of different conifer species^33–36^. The current study is the first endeavor in Kashmir Himalaya elucidating the ectomycorrhization potential of *C*. *nuda* and *C*. *distans* for their prospective use in restoration practices. These fungi were identified on the basis of assessment of their emergence time, color variations, along with the key morphological characteristics of pileus, lamellae, stipe, cortina/veil, annulus, and spores (Supplementary Table S1). The effect of different C and N sources was studied on the mycelial growth of these ECM species under in vitro conditions. Pure cultures of these ECM species were used to study the effect of their inoculation on the seedling growth and survival of *Abies pindrow* (Royle ex D.Don) Royle (Himalayan Pindrow Fir), *Cedrus deodara* (Roxb. ex D.Don) G.Don (Himalayan Cedar), and *Picea smithiana* (Wall.) Boiss. (West Himalayan Spruce). The current study encompasses ecology of hitherto unexplored host-fungus combinations from prized but highly over-exploited Kashmir Himalayan forests.

### Isolation of pure ECM cultures

Fresh and un-infected sporocarps of the two ECM species (*Clitocybe nuda* and *Cortinarius distans*) were collected from different coniferous forests across Kashmir Himalaya (32°20′ to 34°50′ North latitude and 73°55′ to 75°35′ East longitude) during the months of September–October, 2018. Modified Melin-Norkrans (MMN) agar media supplemented with bactericide ‘streptomycin’ was used as a basic medium to isolate pure ECM cultures of the two species. MMN, a standard medium for isolating and maintaining ECM cultures, contains (g/L): 0.5 g KH_2_PO_4_, 0. 25 g (NH4)_2_HPO_4_, 0.15 g MgSO_4_, 0.05 g CaCl_2_, 0.025 g NaCl, 0.012 g FeCl_3_, 0.001 g thiamine hydrochloride, 10 mL hellers micronutrients, 10 g glucose, 4 g malt extract, solidified with 15 g agar powder in 1 L double distilled water^29,37^. The medium pH was adjusted at 5.5–5.8 using 1 N HCL and was autoclaved at a pressure of 15 lb/psi and temperature of 121 °C for 30 min. After sterilization, the medium was cooled to 50–55 °C, supplemented with streptomycin (50 mg/L), and was then poured in sterilized Petri dishes (10 cm diameter).

Surface sterilized (by rectified spirit) healthy sporocarps were cut with sterile surgical blades along the pileal surface to divulge the internal pileal flesh. Three to four small pieces of clean pileal flesh (each of approximately 2 mm size) were inoculated with flame sterilized forceps on to each sterile culture plate containing the medium, under sterile conditions in a laminar flow hood. The inoculated plates were then sealed with parafilm and incubated at 25 °C in the dark for one month. These plates were daily examined for contamination and growth during the first 2 weeks after incubation, and sub-culturing was done for further purification if required. Stock cultures of these ECM species were preserved on MMN slants at 4 °C in a refrigerator until further use.

### Effect of different C and N sources on mycelial growth of ectomycorrhizal fungi under in vitro conditions

The isolated pure ECM cultures of the two ECM species were sub-cultured on broth MMN medium. From the 20–25 days old culture plates (stock cultures), three mycelial-agar inoculum discs (about 0.5 cm diameter) were carefully removed from the actively growing margins of the ECM colonies with a sterilized cork borer under aseptic conditions and placed in one 100 ml screw-capped Erlenmeyer flask containing 50 ml of sterilized MMN medium. This broth MMN medium was supplemented with diverse C and N sources independently, so that C/N ratio remained constant. To analyse utilization of C sources, glucose in the basic culture medium was substituted independently with different C sources like maleic acid, sucrose, and trehalose. The strength of each C source was attuned so that the C concentration in each medium was equivalent to the C concentration of the basic medium. Similarly, to examine the effect of N sources on ECM mycelial growth, di-ammonium hydrogen orthophosphate in the basic culture medium was supplanted with L-alanine, L-asparagine, and potassium nitrate separately. These N sources belong to two categories viz., inorganic N sources (supplied as ammonium-nitrogen and nitrate-nitrogen) and organic N sources (supplied as amino acids). The concentration of each N source was customized so that the N concentration in each medium was equivalent to that of the basic medium. 1 N HCL was used to set the pH of the medium at 5.5. The methodology followed was adopted from Itoo and Reshi^28^.

There were three replicate flasks of each combination of species and C or N source. The loosely capped flasks were incubated for 20 days at a constant temperature of 25 °C in the dark and were shaken at regular intervals. After 20 days, ECM fungi covered the open surface of this broth medium. The contents were harvested and filtered through filter paper (Whatman Grade No. 1), washed with double-distilled water, and the recovered mycelial biomass was transferred to pre-weighed aluminium foil. These were dried in an oven at 60 °C to a constant weight and were then weighed. The mean mycelial growth for each medium, including standard error (SE) was calculated from the values of three replicate flasks. Subsequently, sufficient inoculum was produced for ectomycorrhization of the target host species.

### Effect of ectomycorrhization on conifer seedlings

The host species investigated during the present study included *Abies pindrow*, *Cedrus deodara*, and *Picea smithiana*, which are important constituent elements of the temperate coniferous forests in Kashmir Himalaya^38^. To study the effect of ectomycorrhizal inoculation on growth and survival of *A. pindrow*, *C. deodara*, and *P. smithiana* seedlings, an experiment was set up in the greenhouse at Kashmir University Botanical Garden (KUBG). The soil used for the pot experiment was collected from mature mixed forest of Mammer Kangan, Kashmir, India (34°13′1″ N, 74°59′10″ E at an altitude of 2150 masl) and was double-sterilized in an autoclave for one hour. A preliminary experimentation demonstrated that through this sterilization practice, the soil gets rid of the majority of ECM, pathogenic and soil fungi. Surface-disinfected seeds of the three conifer species were sown in polyethylene bags (size 10 cm × 10 cm) containing 500 g double-sterilized soil. Only one seedling was grown per polybag, and the seedlings were maintained in the greenhouse.

### Ectomycorrhization of seedlings and assessment of their performance

Pure mycelial inoculum acquired from the sporocarps of two ECM species (*Clitocybe nuda* and *Cortinarius distans*) on synthetic medium under sterile conditions was used to study the effect of ectomycorrhizal inoculation on *A. pindrow*, *C. deodara*, and *P. smithiana* seedlings under controlled conditions. Inoculation was performed precisely 3 weeks (21 days) after seed germination. For this purpose, topsoil (2–5 cm) was displaced from the polybags with minimal disruption of the root system, and inoculation was carried out by interspersing 10 ml of mycelia inoculum (i.e., approximately 50 mg) around the roots in the rhizosphere of each conifer seedling using a micropipette. Subsequently, this inoculum was covered with fresh autoclaved forest soil. These seedlings were maintained under standard aseptic conditions (25 ± 2 °C with 60 ± 10% relative humidity) in the greenhouse (closed compartment with electronically controlled conditions) and irrigated as per the need (watered each polybag with 100 ml double distilled water, twice a week) by using sterile syringe. There were ten seedlings of each fungus-host plant combination and uninoculated pots (without any fungal inoculation) served as controls. Additionally, one seedling of each species was excavated every week after ECM inoculation to observe the commencement of ECM-host plant alliance. All the treatments (*C. nuda* inoculated, *C. distans* inoculated and non-inoculated conifer seedlings) were arranged in a completely randomized design.

To evaluate the effect of different ECM isolates on conifer growth, seedlings were carefully excavated and destructively harvested 13 weeks after inoculation. Several plant growth traits like seedling survival, seedling height, number of needles per seedling, root and shoot biomass were measured for all plants in each treatment. Seedling survival was calculated by the number of healthy and green seedlings. Proper care was taken to extricate the roots in intact form. Soil including other extraneous material was circumspectly washed off from the roots with running tap water in the laboratory. These plants were then divided into roots, stems, and needles. Root and shoot dry biomass was assessed after these parts were oven-dried at 60 °C until stable constant dry weight was attained. The dry weight of the stem and needles jointly represented shoot biomass. Moreover, ECM root colonization was observed by examining transverse sections of conifer roots (stained with aniline blue or trypan blue) under a compound microscope for the incidence of ectomycorrhizal morphological structures like emanating hyphae, Hartig net, and mantle.

### Statistical analysis

All statistical analyses were carried out by using the SPSS software (Version 22). Boxplots were plotted for graphically displaying the data distribution through their quartiles in RStudio (Version R 4.0.3). The data were analyzed by one-way analysis of variance (ANOVA) to determine the effect of different C and N sources on the ECM mycelial growth under in vitro conditions and the effect of ectomycorrhization on the studied plant growth traits. The p-values less than 0.05 were regarded as significant. In order to ascertain significant differences among the treatments, means were compared by using Tukey’s HSD post hoc test at *P* < 0.05.

## Results

### Identification and morphological characteristics of ECM fungi

The morphological characteristic features of *Clitocybe nuda* and *Cortinarius distans* fungal sporocarps along with their colony morphology and ECM root morphology are presented in the Supplementary Table S1. Both ECM fungi *C. nuda* and *C. distans* exhibited extremely slow mycelial growth on synthetic MMN media under in vitro settings. Their radial growth stagnated much before covering the entire surface area of the culture plate.

### Effect of different C and N sources on mycelial growth of ectomycorrhizal fungi under in vitro conditions

**Figure 1:**
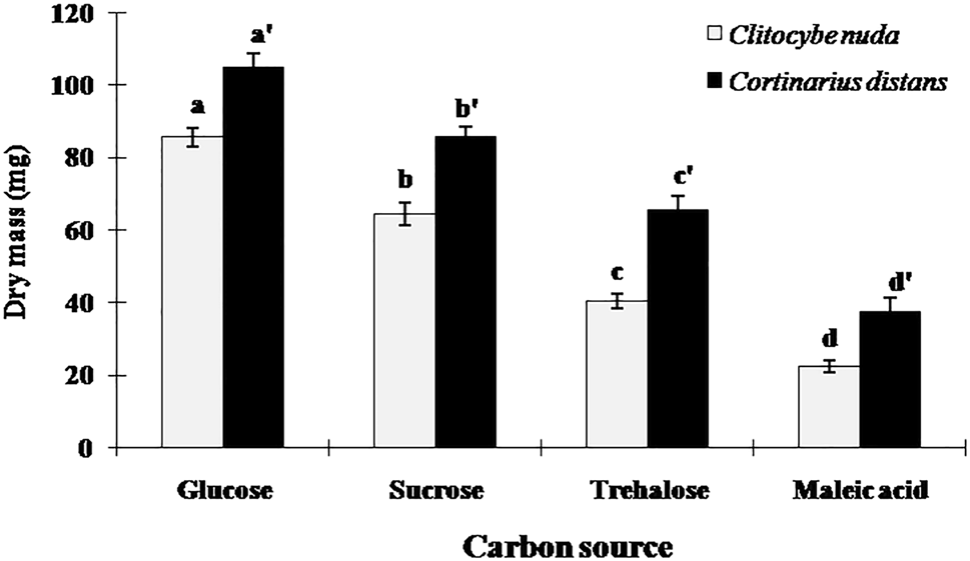
Dry mass (Mean ± SE) of ECM fungi in liquid media containing different C sources. Different letters on the bars indicate that the means are significantly different (Tukey’s HSD test, *P* < 0.05).

The effect of different C and N sources on the mycelial growth of two ECM species viz., *C. nuda* and *C*. *distans* was studied under in vitro conditions. Significant differences were found in ECM mycelial growth amongst the treatments (*P* < 0.05). Both ECM species showed best mycelial growth in media containing glucose, followed by sucrose and trehalose, indicating that glucose is the most suitable C source for the production of well-developed compact mycelial mass of these mycobionts, for their prospective use as inoculum in conifer regeneration projects. However, both species demonstrated reduced growth in medium containing maleic acid as C source (Fig. 1). This suggests that maleic acid is the least preferable C source for these ECM fungi.

**Figure 2:**
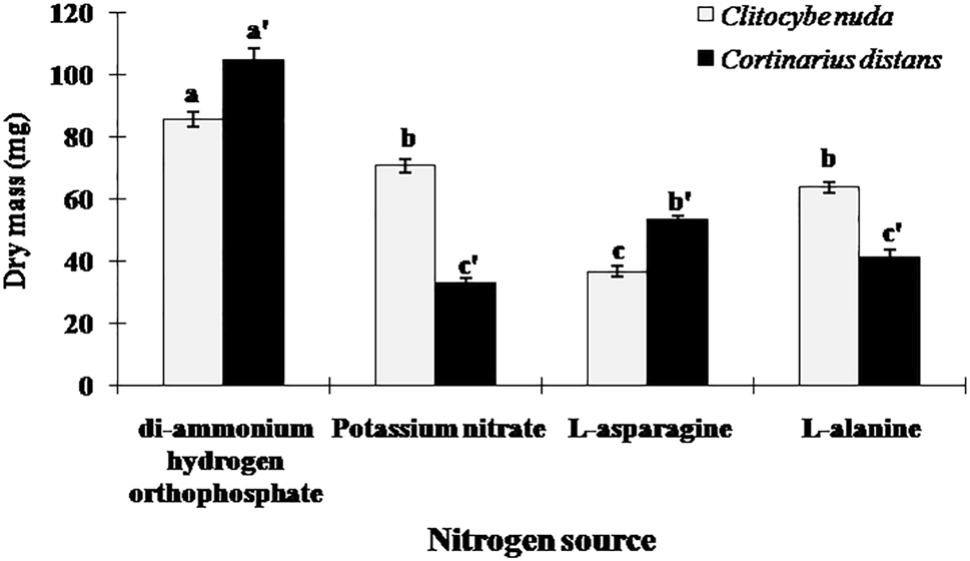
Dry mass (Mean ± SE) of ECM fungi in liquid media containing different N sources.

Both ECM species showed best mycelial growth in MMN medium containing N in ammonium form, indicating that in comparison to other three applied N sources, ammonium is the most preferable N source of these mycobionts. Furthermore, *C*. *nuda* showed higher mycelial growth in medium containing l-alanine than l-asparagine, while as the growth of *C*. *distans* got enhanced in medium containing l-asparagine followed by l-alanine as compared to potassium nitrate (Fig. 2). Thus, mycelial growth of both ECM fungi was higher in media containing inorganic N as compared to media supplemented with organic N.

Both ECM species exhibited optimum growth in basic MMN media (85.62 ± 2.49 mg for *C*. *nuda* and 104.84 ± 3.74 mg for *C*. *distans*). Moreover, mycelial growth of *C. distans* was relatively greater than that of *C. nuda* in the basal media as well as in the media containing different C and N sources. Only exception being the media supplemented with L-alanine and potassium nitrate, which comparatively abridged the growth of *C. distans* but, not that of *C. nuda*.

### Effect of ectomycorrhization on conifer seedlings

**Figure 3:**
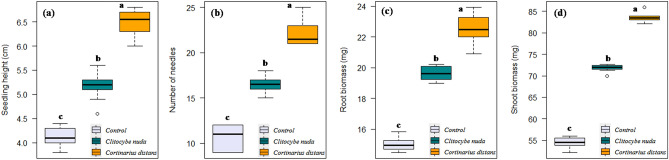
Boxplots showing effect of ECM inoculation on seedling growth traits (**a**) seedling height, (**b**) number of needles, (**c**) root biomass, and (**d**) shoot biomass of *Abies pindrow*. [Box: Tukey box with lower hinge of the box representing first quartile (Q_1_ or 25th percentile), middle horizontal bold line representing median (Q_2_ or 50th percentile), and upper hinge of the box representing third quartile (Q_3_ or 75th percentile); Whiskers: Maximum (Q_4_ or 100th percentile) and minimum (Q_0_ or 0th percentile) values excluding outliers; Small circles: Outlier values; Different letters on the boxes indicate that the means are significantly different (Tukey’s HSD test, *P* < 0.05)].

**Figure 4:**
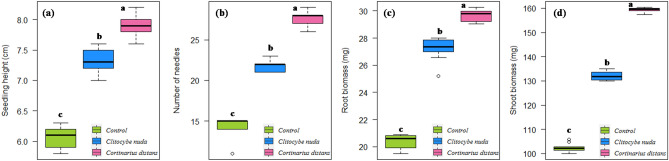
Boxplots showing effect of ECM inoculation on seedling growth traits (**a**) seedling height, (**b**) number of needles, (**c**) root biomass, and (**d**) shoot biomass of *Cedrus deodara*.

**Figure 5:**
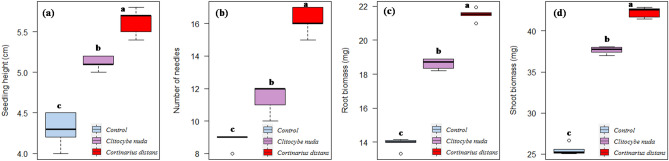
Boxplots showing effect of ECM inoculation on seedling growth traits (**a**) seedling height, (**b**) Number of needles, (**c**) root biomass, and (**d**) shoot biomass of *Picea smithiana*.

All the treated seedlings formed association with the inoculated fungi within 3–4 weeks after inoculation. Roots of control (un-inoculated) conifer seedlings were not colonized by any ECM fungi. Inoculation and colonization of both *C*. *nuda* as well as *C*. *distans* significantly enhanced seedling survival, height, needle number, root and shoot biomass of these conifer species as compared to un-inoculated non-mycorrhizal control seedlings (Table 1). Ectomycorrhization significantly enhanced the survival rate of all host conifer species by 10–15% as compared to their respective control treatments. The height and biomass (especially shoot biomass) of mycorrhizal seedlings were significantly greater (23–36% and 34–40%, respectively) as compared to non-mycorrhizal seedlings. Moreover, inoculation of target conifer species with *C*. *distans* proved significantly more effective over all other treatments (Figs. 3, 4, and 5).

**Table 1:** Effect of ECM inoculation on seedling growth traits (mean ± SE) in *A. pindrow*, *C. deodara*, and *P. smithiana* under greenhouse conditions.

Treatment	Seedling growth traits (110-days-old seedlings)				
	Seedling survival (%)	Seedling height (cm)	Needle no	Root biomass (mg)	Shoot biomass (mg)
***A. pindrow***					
Control	70	4.11^c^ ± 0.06	10.6^c^ ± 0.40	15.04^c^ ± 0.13	54.48^c^ ± 0.40
*Clitocybe nuda*	80	5.16^b^ ± 0.08	16.6^b^ ± 0.31	19.62^b^ ± 0.15	71.83^b^ ± 0.25
*Cortinarius distans*	85	6.50^a^ ± 0.08	22.0^a^ ± 0.42	22.58^a^ ± 0.28	83.55^a^ ± 0.32
***C. deodara***					
Control	60	6.08^c^ ± 0.05	14.4^c^ ± 0.40	20.41^c^ ± 0.15	102.39^c^ ± 0.57
*Clitocybe nuda*	75	7.30^b^ ± 0.05	21.8^b^ ± 0.20	27.19^b^ ± 0.26	132.08^b^ ± 0.53
*Cortinarius distans*	75	7.90^a^ ± 0.05	27.6^a^ ± 0.26	29.65^a^ ± 0.13	159.27^a^ ± 0.32
***P. smithiana***					
Control	50	4.30^c^ ± 0.09	8.8^c^ ± 0.20	13.91^c^ ± 0.14	25.52^c^ ± 0.29
*Clitocybe nuda*	55	5.12^b^ ± 0.03	11.4^b^ ± 0.40	18.61^b^ ± 0.14	37.65^b^ ± 0.19
*Cortinarius distans*	60	5.62^a^ ± 0.07	16.2^a^ ± 0.37	21.49^a^ ± 0.15	42.26^a^ ± 0.28

Mean values between rows (treatments) separated by different letters are significantly different (*P* < 0.05) according to Tukey’s test.

Significant differences were found in all the studied growth parameters among the treatments (*P* < 0.05). Thus, this study ascertained *C*. *distans* and *C*. *nuda* as efficient inoculants for augmentation of conifer survival and growth for their potential use as planting material in restoration projects.

## Discussion

*Cortinarius distans* exhibited higher growth than *Clitocybe nuda* across all the treatments (Fig. 1) and proved considerably more efficient in enhancing the survival and growth of all the studied conifer host species (Table 1). This signifies that the former ECM mycobiont might be producing more expanded hyphal networks in soil/roots under natural conditions, thus conferring a rational advantage to the host plant growth in nutrient-poor soil conditions by facilitating nutrient access and uptake, which otherwise could have been relatively distant from the host plant roots and thus inaccessible to the host plant. Hence, this comparatively better-growing ECM species can be employed for large-scale mycelial inoculum production and subsequent inoculation of conifer seedlings.

The present study demonstrated that the ECM mycobionts could utilize a broad spectrum of C and N sources for growth in pure culture. This capability to exploit specific simple sugars as C sources, independent of their host plants, point to their facultative symbiotic nature^39^. The basic MMN medium (containing glucose and di-ammonium hydrogen orthophosphate as C and N sources respectively) was the most preferred medium that resulted in the optimum mycelial growth of these fungal symbionts. The current study signifies that ECM fungi prefer inorganic N (primarily ammonium) over organic N under in vitro conditions, which has also been confirmed by several workers with a different array of ECM fungi, both under field and laboratory settings (See^25–29,31,37,40^).

Preferential utilization of ammonium (NH_4_^+^) over all other applied N treatments has been attributed to the presence of ammonium-assimilating enzymes in these symbionts^41,42^. Moreover, ammonium utilization is an energy-efficient process, as ammonium is directly incorporated into the fungal metabolites, while nitrate and other organic compounds require enzymatic conversion to ammonia prior to assimilation, which entails additional energy expenses^37,43^. Contrary to this, few studies have reported ammonium as the least preferred N source of ECM fungi^44^. Thus, the selection of apposite N source is critical for optimizing the inoculum production of specific ectomycorrhizal fungi.

The nitrate (NO_3_^-^) utilization ability of ECM fungi is extremely erratic (either excellent or poor) among the different species^29,45^. Potassium nitrate, being an inorganic N source, should augment the growth of ECM fungi when compared to organic N sources, but in our study, nitrate N considerably abridged the mycelial growth of *C. distans*, signifying the probability of an inhibitory effect of nitrate on its growth. With consistency to our observation, the growth of several other ECM fungi has been shown to be negatively affected by the incidence of nitrate, both in vitro as well as in soil^43,46^.

In the present study, the tested ECM fungi also utilized amino acids like alanine and asparagine in pure culture, which implies that they may support host plant growth in the acidic organic soil by facilitating uptake and utilization of organic compounds. For ECM fungi, amino acids can function not only as N sources, but also as C sources^29^, implying that this heterotrophic C assimilation from organic compounds may endure some of the C cost entailed in ectomycorrhizal infection^25^.

This varied capability to utilize diverse C and N sources function as imperative ecophysiological markers of ECM fungi since it is species or even strain-specific and reasonably dependent on the type of culture media and culture conditions^26,28,29,44^. This could probably be correlated to the disparate fungal endurance in the soil, their advanced adaptation strategies in occupying different soil microsites of varied nutrient status, their efficient colonization tactics, and their distribution pattern^26,29^.

Our results revealed that both *C*. *nuda* and *C*. *distans* formed ectomycorrhizal association with the roots of the three Himalayan conifer seedlings and significantly enhanced their survival, growth and biomass productivity. However, both the tested ECM fungi varied significantly in their performance augmenting capability, with *C. distans* being more effective, suggesting that this ECM species is ideal for mass inoculum production intended for prospective large-scale inoculation of experimental, nursery, and/or field conifer plantations.

The positive correlation between ectomycorrhization and growth traits of host plants that engross numerous physical, biochemical, physiological, and molecular amendments has been attributed explicitly to the extended tertiary root system, increased absorption and transport of water by extensive extraradical ECM mycelia, facilitated acquisition of nutrients (predominantly N, C and P content), improved photosynthetic capability, production of growth regulators, phytohormone signalling, stress tolerance, attraction of other plant growth promoting microbes to ectomycorrhizosphere and stimulation of defence mechanisms^30,47^.

The forests of Kashmir Himalaya are prone to intense fires, particularly during autumn. Fire-induced alteration in forest soil properties and diminished ectomycorrhizal communities can persist for decades^47,48^, severely impairing the natural forest regeneration. Additionally, the establishment of newly planted seedlings in these areas depends on the prevailing fraction of ECM fungi retrieved, if any, after fire. Therefore, large-scale ectomycorrhization of these seedlings before out-planting on severely blazed areas is recommended for their successful restoration^47^. Maintenance and restoration of structural organization and functional integrity of dominant coniferous forest ecosystems through ECM-mediated restoration efforts would not only help conserve biodiversity but would also improve all vital ecosystem services^15^. Although the findings of our study are restricted to specific host-fungus combinations, these could potentially hold true for diverse ECM fungi and many other economically important host tree species of Kashmir Himalaya.

## Conclusion

The present study is a preliminary investigation carried out to appraise the potential role of ectomycorrhizal biotechnology in conifer regeneration in Kashmir Himalaya. Ectomycorrhizal fungi (*Clitocybe nuda* and *Cortinarius distans*) exhibited the capability to utilize a wide spectrum of C and N sources for growth under in vitro culture conditions. The basic MMN medium consisting of glucose (C) and ammonium (N) was the most preferred medium of these fungal symbionts. Our results further revealed that both *C*. *nuda* and *C*. *distans* significantly enhanced the survival and improved growth and productivity of *A. pindrow*, *C. deodara*, and *P. smithiana* seedlings. However, *C. distans* proved significantly more effective, signifying that this comparatively better-growing ECM species is ideal for mass inoculum production intended for prospective large-scale ectomycorrhization of conifer plantations in Kashmir Himalaya. Even though the findings of our study are restricted to specific host-fungus combinations, these could also hold true for diverse ECM fungi and many other economically important host tree species. In summary, the present study paved the way for identification and inoculum production of suitable ECM symbionts of dominant conifers of Kashmir Himalayan forests for their use in raising seedlings with high survival rate and improved growth traits, which would prove very efficacious for large-scale ectomycorrhization of conifer seedlings for their potential use in the restoration of large tracts of degraded forest ecosystems.

## Supplementary Information

Supplementary Information.

## Data Availability

Appropriate permission was obtained for the collection of conifer host plant material from different forest ecosystems of Kashmir Himalaya. The voucher specimens of *Abies pindrow*, *Cedrus deodara*, and *Picea smithiana* were deposited in Kashmir University Herbarium (KASH) at the Centre of Biodiversity and Taxonomy (CBT) of the Department of Botany under the voucher specimen numbers 2829-(KASH), 2830-(KASH), and 2831-(KASH), respectively which were identified by the curator Mr. Akhtar H. Malik. The study complies with all local, national, and international guidelines. All the data generated or analysed in the current study have been incorporate in this manuscript and its supplementary file.
