# Supplementary material for: Seedling ectomycorrhization is central to conifer forest restoration: a case study from Kashmir Himalaya
Source: Sci Rep. 2022 Aug 3;12:13321. doi: 10.1038/s41598-022-17073-7 (PMC9349292; doi:10.1038/s41598-022-17073-7)
Supplement: Supplementary file 1 — Supplementary Information. [file 41598_2022_17073_MOESM1_ESM.docx]

**Additional information**

**Seedling ectomycorrhization is central to conifer forest restoration: A case study from Kashmir Himalaya**

Rezwana Assad*, Zafar Ahmad Reshi and Irfan Rashid

Department of Botany, University of Kashmir, Srinagar-190006, Jammu and Kashmir, India

***Corresponding author**

Rezwana Assad

Department of Botany,

University of Kashmir, Hazratbal, Srinagar, Jammu and Kashmir 190006

E-mail Id: [rezumir@gmail.com](mailto:rezumir@gmail.com)

**Supplementary Table S1.** Morphological characteristics of the studied ECM fungi.

| **ECM Fungi** | ***Clitocybe nuda* (Bull.) H.E. Bigelow & A.H. Sm.** | ***Cortinarius distans* Peck** |
| --- | --- | --- |
| **Family** | Tricholomataceae | Cortinariaceae |
| **Synonymy** | **7 synonyms**  *Agaricus nudus* Bull.,  *Cortinarius nudus* (Bull.) Gray,  *Lepista nuda* (Bull.) Cooke,  *Tricholoma nudum* (Bull.) P. Kumm.,  *Gyrophila nuda* (Fr.) Quél.,  *Rhodopaxillus nudus* (Bull.) Maire, and  *Tricholoma personatum* var. *nudum* (Bull.) Rick [49-51] | **1 synonym**  *Phaeomarasmius distans* (Peck) Singer  [51-53] |
| **General description** | *Clitocybe nuda*, commonly known as ‘Blewit’, is a potential ectomycorrhizal fungus [33,35,36,54] that grows in clusters in the coniferous forests of Kashmir Himalaya. It appears in autumn, around in the month of September. One of the diagnostic features of this mushroom is transformation of color, from being conspicuous pale purple colored at young stage to turning brownish/ beige at maturity. | *Cortinarius distans* is an ectomycorrhizal mushroom [34,55], found in association with conifers in Kashmir Himalayan forests. It is brownish in color, appears around September, and is difficult to identify species with rare distribution. However its diagnostic features include distantly spaced gills, grainy cap structure, and whitish ring zone. |
| **Pileus** | Cap 4-20 cm broad; broadly convex shaped with incurved margin at young stage, turning flat with an uplifted and wavy margin at maturity; smooth and glabrous surface, faintly sticky when moist; purplish in color when young, transform to dull brownish/beige towards maturity, often with lighter margin; flesh soft, thick, dull lilac initially and turn whitish at maturity. | Cap 2-7 cm broad; bell-shaped to rounded; light grainy or scaly texture; orangish-brown colored when young, color changes to orangish-tan at maturity; whitish margin initially, splitting with age; flesh white when young, turn brownish and flaky with age. |
| **Lamellae** | Gills attached to the stipe, close and crowded; lilac at young stage and turn pinkish-buff to brownish with age. | Gills distantly spaced, yellowish brown when young, become cinnamon brown on maturity; affixed to the stipe by a notch; at the outset enveloped with a white cortina (veil). The presence of cortina/veil between pileus and stipe which protects gills at immature stage and then disappears with age, is a distinctive attribute of the species belonging to genus *Cortinarius*. |
| **Stipe** | Stipe 3-10 cm long, apex 1-3 cm wide; equal in diameter or bulbous at the base; dry; slightly hairy, covered with white fibrils, slight scaly textured at apex; pale purplish in color, appear lighter than the cap, turn brownish at maturity; solid, pale brown colored inside. | Stipe 4-8 cm long, about 1.5cm thick; equal in diameter or club-shaped initially when young, turn cylindrical on maturity; light silky texture; brownish in color, white near apex when young; generally with a white annulus (ring zone); whitish basal mycelium. |
| **Spores** | Spores are ellipsoid; spore print appears pale pinkish. | Spores are ellipsoid; spore print appears rusty brown. |
| **Odour** | Odour pleasant, slightly fragrant. | **Odour**: Odour radish-like or non-distinctive. |
| **Pileus** | **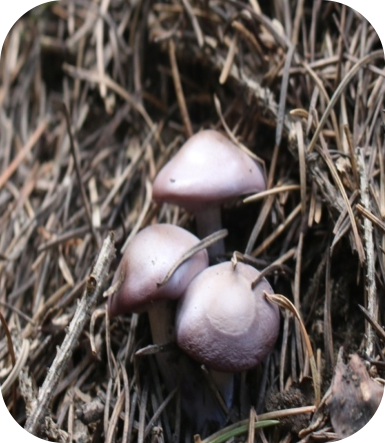** | **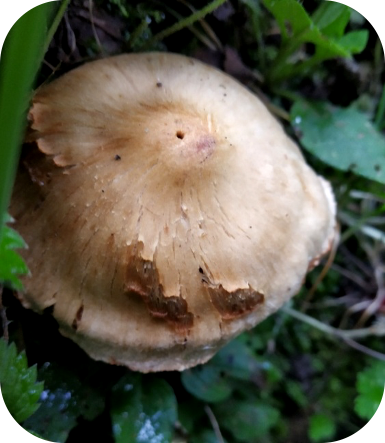** |
| **Stipe and Lamellae** | **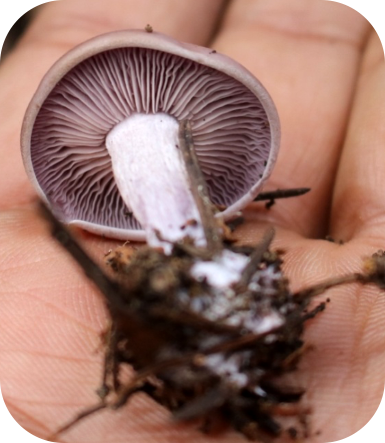** | **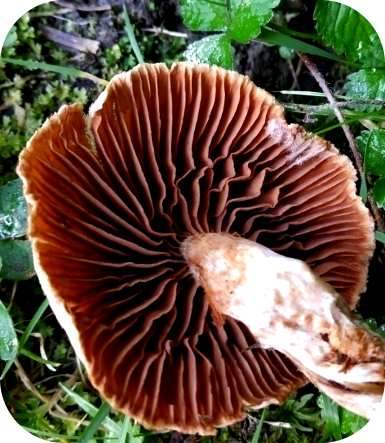** |
| **ECM pure culture** | **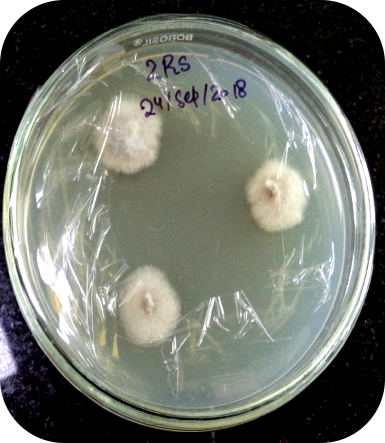** | **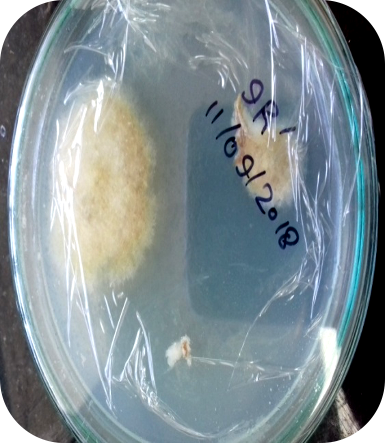** |
| **Colony morphology** | Fungal colonies of *C*. *nuda* appeared light purplish in color; velvety in texture with compact mycelia; round shaped with regular margin. | *C*. *distans* fungal colonies appeared beige; cottony in texture with airy and loosely interwoven mycelia; white colored regular margin. |
| **ECM root morphology** | Tertiary roots of host plants colonized by *C. nuda* appeared beige with creamish apices; unbranched and dichotomous ramification patterns; mantle covered with whitish mycelium. | Plant roots infected with *C. distans* appeared dark brown in color with irregularly pinnate ramification pattern; well developed mantle covered with beige cottony mycelium. |
